# Long-term neuromuscular alterations during botulinum toxin treatment for chronic migraine

**DOI:** 10.1016/j.cnp.2026.06.010

**Published:** 2026-06-22

**Authors:** Tina Plensäll, Maarika Liik, Artor Pogosean, Annika Eriksson, Malin Garrett, Jan Olszowy, Sven Jackmann, Anna Rostedt Punga

**Affiliations:** a Neurology, Department of Medical Sciences, Uppsala University, Uppsala, Sweden; b Neurology, Uppsala University Hospital, Uppsala, Sweden; c Clinical Neurophysiology, Uppsala University Hospital, Uppsala, Sweden; d Clinical Neurophysiology, Department of Medical Sciences, Uppsala University, Uppsala, Sweden

**Keywords:** Botulinum toxin, Neuromuscular transmission, Chronic migraine, Myopathy, Denervation

## Abstract

**Objective:**

To evaluate early and long-term neuromuscular and clinical effects of botulinum toxin type A (BoNTA) in women with chronic migraine undergoing sustained treatment.

**Methods:**

Forty female patients were stratified by cumulative BoNTA exposure (treatment-naïve, ∼1 year, 2–5 years, >5 years). Clinical outcomes included migraine frequency, pain burden, and quality of life. Neurophysiological assessments during one treatment cycle comprised motor nerve conduction studies, quantitative EMG, jitter analysis, and blink reflex. Serum neurofilament light chain (NfL) and glial fibrillary acidic protein (GFAP) were also measured.

**Results:**

BoNTA significantly reduced migraine days (*p* = 0.0008) with sustained benefit across treatment groups. Neuromuscular side effects were reported in 75% of patients treated for ≥1 year, including ptosis, diplopia, and shoulder weakness. In treatment-naive patients, frontalis CMAP amplitudes declined significantly from baseline to week 12 (*p* = 0.0006), whereas cumulative reductions were most pronounced in the trapezius muscle in patients treated for 2–5 years (*p* < 0.0001). EMG showed spontaneous activity consistent with denervation in up to 60% of patients and myopathic patterns in 30–40% of long-term-treated individuals. Abnormal jitter in the orbicularis oculi was present in 47% at week 12. NfL levels were higher in long-term treated groups at week 4 (*p* = 0.0095), while GFAP remained unchanged. Subtle blink reflex abnormalities suggested possible central modulation.

**Conclusions:**

BoNTA is associated with persistent neuromuscular alterations, with early effects in facial muscles and cumulative changes in the trapezius.

**Significance:**

These findings support the occurrence of treatment-dependent neuromuscular side effects and highlight the need for monitoring during long-term therapy.

## Highlights

• Neuromuscular side effects affect up to 75% with chronic Botulinum toxin A (BoNTA).
• Long-term BoNTA results in persistent denervation, increased jitter, and myopathy.
• Serum Neurofilament Light rises during prolonged BoNTA treatment.

## 1 Introduction

Botulinum toxin type A (BoNTA) exerts its primary pharmacological effect by inhibiting acetylcholine release at the presynaptic terminal(Blasi et al., 1993), resulting in localized chemodenervation, flaccid paralysis, and, with repeated administration, muscle atrophy(Koerte et al., 2013). Its clinical use has expanded markedly over the past decades, encompassing therapeutic indications, such as chronic migraine and dystonia, as well as cosmetic procedures, in which facial and cervical muscle injections are among the most frequently performed non-surgical interventions worldwide(Monash et al., 2025).

Beyond its neuromuscular actions, BoNTA also modulates sensory processing and pain pathways, contributing to its therapeutic efficacy in headache disorders. Injections into facial muscles can influence trigeminal and facial nerve functions, and neurophysiological studies demonstrate changes in brainstem excitability, including altered blink reflex parameters and nociceptive processing(Kurtkaya Kocak and Bora Tokcaer, 2025; Zuniga et al., 2008). Adverse effects are typically mild and localized, such as injection-site pain, bruising, or edema. However, diffusion into adjacent musculature may lead to neuromuscular symptoms(Punga et al., 2023), including dysarthria, dysphagia, blepharoptosis, or difficulty opening the eye, which are generally dose- and site-dependent (Sethi et al., 2021). Spread to contralateral or distal muscles has also been documented, including upper-limb involvement following facial injections(Punga et al., 2015). Notably, BoNTA-induced neuromuscular transmission impairment may mimic conditions such as myasthenia gravis or motor neuron disease, posing diagnostic challenges(Punga and Liik, 2020).

Although BoNTA is FDA-approved for the temporary treatment of glabellar lines, its use frequently extends beyond this indication. The visible clinical effect usually lasts 3–4 months, yet neurophysiological studies indicate that subclinical abnormalities in injected and neighboring muscles may persist far longer(Punga et al., 2016). Facial and bulbar muscles, characterized by relatively low expression of muscle-specific tyrosine kinase (MuSK), may be particularly vulnerable to prolonged denervation(Punga et al., 2011). Despite its widespread adoption and frequent long-term administration across diverse clinical populations, the extended safety profile of cumulative BoNTA exposure remains insufficiently understood.

BoNTA was approved for chronic migraine in 2010 following the pivotal PREEMPT phase III trials, which demonstrated significant reductions in headache frequency, pain intensity, and acute medication use, as well as improved quality of life(Dodick et al., 2010). Current European Headache Federation guidelines recommend repeated intramuscular administration of 155–195 units across 31–39 injection sites every 12 weeks (Bendtsen et al., 2018). Initially, thought to act via muscle relaxation and vascular decompression (Binder et al., 2000), BoNTA is now recognized to exert its therapeutic effects mainly through sensory modulation. Proposed mechanisms include inhibition of pro-inflammatory neurotransmitter and neuropeptide release, disruption of SNARE-mediated exocytosis, and reduced insertion of pain-sensitive ion channels(Burstein et al., 2020). Increasing evidence also suggests potential central effects via anterograde axonal transport and transcytosis (Restani et al., 2011).

Alongside clinical neurophysiological assessments of neuromuscular function, biomarkers such as neurofilament light chain (NfL) and glial fibrillary acidic protein (GFAP) have been proposed as indicators of neuroaxonal injury. NfL is released into cerebrospinal fluid and blood following central or peripheral nervous system damage and is elevated across a spectrum of neurodegenerative conditions.

This prospective study aimed to characterize both early and long-term neuromuscular, clinical, and biochemical consequences of BoNTA treatment in patients with chronic migraine. We hypothesized that sustained treatment for more than two years would be associated with persistent atrophy in the injected muscles.

## 2 Methods

### 2.1 Study design, ethical approval, and eligibility criteria

This prospective observational study was approved by the Swedish Ethical Review Authority (Dnr 2023–00829-01; approval date 2023-03-13). Written informed consent was obtained from all participants diagnosed with chronic migraine.

Inclusion criteria were:

1. a diagnosis of chronic migraine according to ICD-10 (G43.3 and/or G43.7), and
2. ongoing or planned BoNTA treatment at the neurology outpatient clinic of Uppsala University Hospital.

To reduce sex-related variability in electrophysiological measurements, and because the clinic's BoNTA-treated chronic migraine population is predominantly female (91%), only women were enrolled. Exclusion criteria comprised previous BoNTA exposure for non-migraine indications (e.g., cosmetic treatment) and any known neuromuscular disorder (e.g., neuropathy, myopathy, myasthenia gravis).

### 2.2 Chronic migraine treatment protocol and patient stratification

BoNTA was administered according to the standardized PREEMPT (Phase III REsearch Evaluating Migraine Prophylaxis Therapy) protocol (Aurora et al., 2010; Diener et al., 2010), using a fixed-site, fixed-dose injection paradigm. Each treatment cycle consisted of 155 units of onabotulinumtoxinA (Botox®), distributed across 31 predefined injection sites involving seven head and neck muscle groups:

• Corrugator muscles: 5 U per site, 2 sites (total 10 U)
• Procerus muscle: 5 U at 1 site (total 5 U)
• Frontalis muscles: 5 U per site, 4 sites (total 20 U)
• Temporalis muscles: 5 U per site, 8 sites (total 40 U)
• Occipitalis muscles: 5 U per site, 6 sites (total 30 U)
• Cervical paraspinal muscles: 5 U per site, 4 sites (total 20 U)
• Trapezius muscles: 5 U per site, 6 sites (total 30 U)

Additional optional “follow-the-pain” injections were administered according to individual symptomatology, most commonly to the masseter muscle (5–10 U per side), and were given in approximately 50% of patients.

BoNTA treatments were administered at regular intervals, typically every 12 weeks, in accordance with standard clinical practice. Importantly, in relation to the electrophysiological assessments performed in this study, the frontalis and trapezius muscles were included in the standard PREEMPT injection protocol and were therefore repeatedly exposed to BoNTA, whereas the nasalis and orbicularis oculi muscles were not injected, thereby serving as non-injected reference muscles.

Participants were stratified into four groups according to cumulative duration of BoNTA treatment and corresponding exposure to repeated treatment cycles:

Group 1: treatment-naive patients initiating BoNTA during the study period (no previous treatment cycles).

Group 2: patients treated for approximately 1 year (approximately 4–5 treatment cycles).

Group 3: patients treated for 2–5 years (approximately 8–22 treatment cycles).

Group 4: patients treated >5 years (more than 22 treatment cycles).

Clinical and electrophysiological outcomes were compared across groups.

### 2.3 Assessment of migraine burden, side effects, and quality of life

Migraine burden was assessed using a structured headache diary in which participants recorded headache frequency, pain intensity using a Visual Analogue Scale (VAS), and associated migraine symptoms such as photophobia, phonophobia, nausea, and functional limitations. Health-related quality of life was evaluated using validated instruments from the Swedish Headache Registry, including the EuroQol five-dimension scale (EQ-5D-5L).

Adverse events, with specific attention to potential BoNTA-related neuromuscular side effects (ptosis, dysphagia, diplopia, blurred vision, shoulder or neck weakness), were recorded by the treating headache nurse (T.P.).

### 2.4 Electrophysiological assessments

All electrophysiological examinations were performed using Nicolet EDX equipment (Natus®). In treatment-naïve patients (group 1), baseline measurements were obtained prior to the first BoNTA injection, followed by repeated assessments at weeks 1, 4, 8, and 12. In previously treated patients (groups 2–4), neurophysiological examinations were performed at weeks 1, 4, 8, and 12 within an ongoing treatment cycle following a scheduled injection.

These time points were selected to cover one complete 12-week treatment cycle, with week 12 corresponding to the time point immediately preceding the next planned injection. Electrophysiological investigations focused on muscles with different exposure to BoNTA treatment (injected vs non-injected), allowing assessment of both local and potential systemic effects.

#### 2.4.1 Motor nerve conduction studies

Motor nerve stimulation was performed bilaterally:

• **Facial nerve**: stimulated below the ear; recordings obtained from the frontalis (injected muscle) and nasalis (non-injected muscle).
• **Accessory nerve**: stimulated posterior to the sternocleidomastoid muscle; recordings obtained from the trapezius muscle (injected muscle).

Compound muscle action potential (CMAP) amplitude and duration were measured.

#### 2.4.2 Quantitative electromyography (QEMG)

Bilateral QEMG was performed in the frontalis and trapezius (injected muscles) as well as masseter (injected in 50%) using a concentric needle electrode (26G; 38 × 0.45 mm; Ambu® Neuroline, Denmark). The orbicularis oculi (non-injected muscle) was examined bilaterally using a smaller concentric needle electrode (30G; 25 × 0.3 mm; Dantec® DCN, Natus, Ireland). Analyzed parameters included spontaneous activity (denervation), motor unit potential (MUP) amplitude, duration, polyphasia, and interference pattern during slight and maximal voluntary contraction.

### 2.5 Concentric needle jitter (CNE) analysis

Concentric needle jitter analysis (CNE) of the left orbicularis oculi muscle was performed using a disposable concentric needle electrode (30G; 25 × 0,3 mm; Dantec® DCN, Natus; Ireland). Jitter was quantified as mean consecutive difference (MCD) in microseconds, derived from up to 20 acceptable potential pairs. Recordings were obtained using standard filter settings (1000 Hz–10 kHz). Reference limits were defined according to locally established normative data for orbicularis oculi mean MCD: ≤ 35 μs and for individual fiber pair jitter: MCD ≤ 45 μs

#### 2.5.1 Blink reflex testing

Blink reflexes were elicited by electrical stimulation of the supraorbital and infraorbital trigeminal branches, with bilateral recordings from the orbicularis oculi.

Supraorbital stimulation generates the early R1 response, mediated through a pontine oligosynaptic trigeminal–facial circuit, whereas both stimulation sites elicit the bilateral R2 responses, mediated via polysynaptic interneurons in the medullary spinal trigeminal nucleus.

Abnormal responses were defined as:

• R1 latency > 12 ms or absent R1 → indicating dysfunction within the peripheral trigeminal/facial pathways and/or the pontine interneuronal circuit.
• R2 latency > 35 ms or absent R2 (for both supraorbital and infraorbital stimulation)  → indicating involvement of the brainstem polysynaptic pathways and/or peripheral trigeminal–facial conduction(Gunduz et al., 2024; Jaaskelainen, 1995).

### 2.6 Serum neurofilament light (NfL) and glial fibrillary acidic protein (GFAP)

Serum NfL and GFAP concentrations were measured using the Quanterix Simoa® Neuro 2-Plex B Advantage Kit (Quanterix Corporation, Billerica, MA, USA) at weeks 1, 4, and 12. Quality-control samples were included in each assay run. Reference values were based on previously published data (Lanz et al., 2025):

• NfL: mean 8.1 pg/mL (range 2.9–37.2 pg/mL)
• GFAP: mean 101.4 pg/mL (range 40.1–299 pg/mL).

### 2.7 Statistical analysis

All statistical analyses were conducted using GraphPad Prism version 10.6.1. Data distribution was assessed using the D'agostino-Pearson omnibus normality test. Because electrophysiological variables were nonparametric, the Wilcoxon matched-pairs signed-rank test was used to compare electrophysiological parameters. Between-group comparisons were conducted using the Mann-Whitney *U* test. To improve interpretability and reduce variability, final analyses were performed on pooled data (mean values) from the left and right sides of each muscle. A *p*-value ≤0.05 was considered statistically significant.

## 3 Results

### 3.1 Demographics, migraine frequency and burden, and side effects

Forty women with chronic migraine were included (mean age 43.5 ± 12.8 years). Demographic characteristics are presented in Table 1.

**Table 1:** Demographic data, including migraine characteristics, electrophysiological characteristics, and neuromuscular side effects, for the cohort.

	Group 1 (*N* = 10)	Group 2 (*N* = 7)	Group 3 (*N* = 14)	Group 4 (*N* = 9)
**Age** mean ± SD (range)	36 ± 10 (24–59)	40 ± 10 (29–53)	44.5 ± 13 (24–68)	53 ± 12.5 (35–78)
**Duration migraine** mean ± SD (range)	11 ± 7.5 (2−23)	17.5 ± 15 (3–38)	15.5 ± 13.5 (4–48)	20 ± 12 (8–37)
**Duration BoNTA** mean ± SD (range)	0 (0–0)	0.9 ± 0.5 (0.5–1.5)	3.5 ± 0.9 (2–5)	7.1 ± 1.7 (5–10)
**Migraine days during the study period** mean ± SD (range)	40.6 ± 12.4 (21.0–58.0)	21.7 ± 14.1 (6.0–40.0)	20.8 ± 16.0 (3.0–56.0)	15.2 ± 7.4 (10.0–27.0)
**Neuromuscular side effects (%)**	10%	100%	69%	67%
**Myopathy on EMG (%)** Frontalis Trapezius Orbicularis oculi	**N = 10** 0 (0%) 0 (0%) 0 (0%)	***N* = 6** 1 (17%) 0 (0%) 0 (0%)	**N = 13** 5 (38%) 4 (31%) 2 (15%)	**N = 9** 4 (44%) 3 (33%) 4 (44%)

Neuromuscular side effects include the following symptoms: ptosis, diplopia, shoulder weakness, difficulty swallowing, and/or difficulty chewing.

**Fig. 1:**
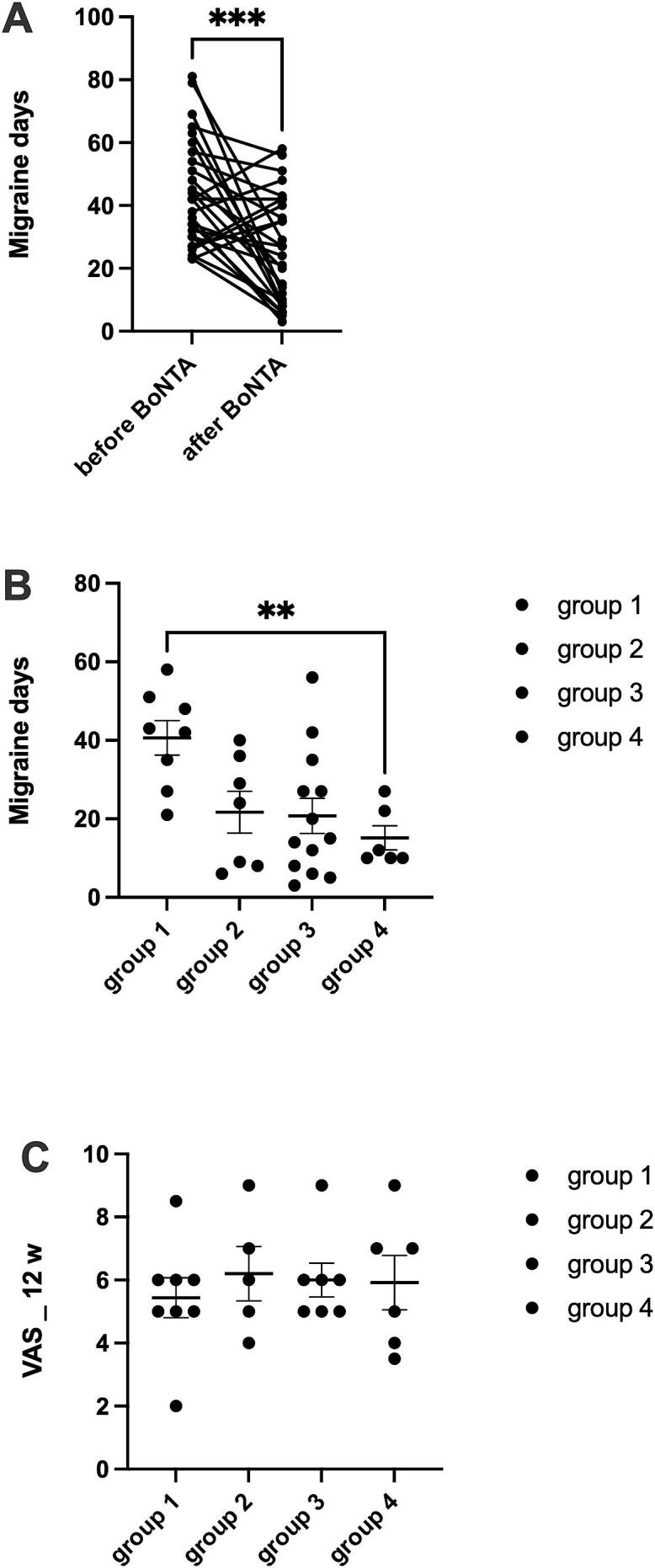
Diary-based migraine frequency and pain intensity before and during BoNTA treatment. (A) Self-reported migraine days per 12 weeks before initiation of BoNTA therapy and during treatment. (B) Migraine days recorded during the 3-month study period, stratified by treatment-duration groups. (C) Pain intensity was measured using the Visual Analog Scale (VAS). Statistical significance: **p ≤ 0.01; ***p **≤** 0.001.

Migraine diary data were available for 28 participants. Prior to BoNTA initiation, the median number of migraine days per 12 weeks was 42 (IQR 30–56), decreasing to 25.5 days (IQR 9–41.5) during treatment. The Wilcoxon matched-pairs signed-rank test confirmed a significant reduction (*p* = 0.0008; Fig. 1A). Across treatment-duration groups, ANOVA demonstrated significant differences in migraine days (*p* = 0.0186), with treatment-naïve patients (group 1) reporting more migraine days than groups 2–4 (Fig. 1B). No differences were observed among groups 2, 3, and 4.

Pain intensity (VAS) did not differ significantly between groups (median 5.5–6; *p* = 0.9315; Fig. 1C). EQ-5D-5L total and pain-domain scores at week 12 also showed no group differences (*p* = 0.4907 and *p* = 0.7650). EQ-5D-5L scores remained relatively stable. In group 1, scores ranged from 0 to 9 at baseline and 0 to 6 at week 12. In groups 2–4, scores ranged from 0 to 10 at week 1 and from 0 to 9 at week 12.

Side-effect data were available for 33 participants. Reported neuromuscular symptoms included ptosis (*n* = 13; 39%), shoulder weakness (*n* = 10; 30%), swallowing/chewing difficulties (*n* = 4; 12%), and diplopia (n = 4; 12%). Only one participant in group 1 (10%) experienced any of these side effects, compared with 12 participants (75%) in groups 2–3 and 6 participants (75%) in group 4.

### 3.2 Serum GFAP and neurofilament light chain (NFl)

Serum GFAP and NfL were measured at weeks 1, 4, and 12 following BoNTA injection.

**Fig. 2:**
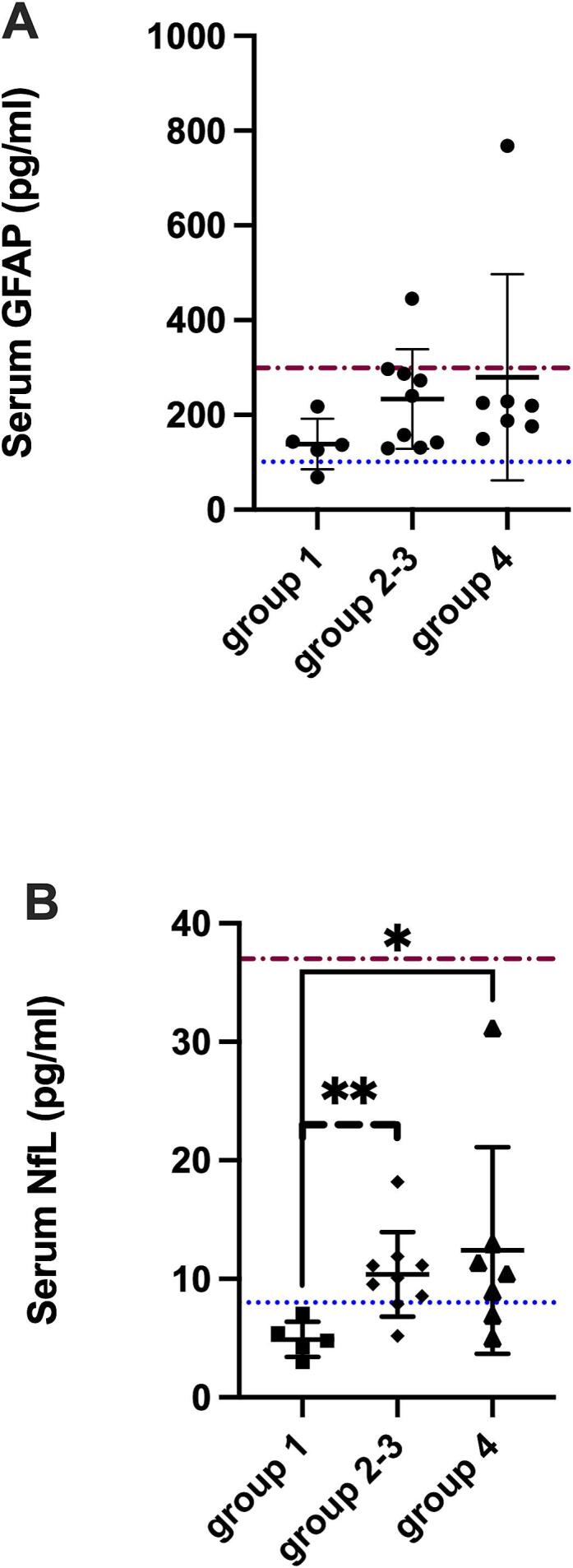
Serum neuroaxonal and glial markers following BoNTA treatment. (A) Glial fibrillary acidic protein (GFAP) and (B) neurofilament light chain (NfL) levels measured in serum (pg/mL) at week 4 post-injection using Quanterix Simoa technology. The blue dotted line indicates the reference mean, and the red dotted line denotes the upper limit of normal based on previously published healthy control data [15]. (For interpretation of the references to colour in this figure legend, the reader is referred to the web version of this article.)

GFAP levels did not differ significantly between groups at any time point (week 1: *p* = 0.5793; week 4: *p* = 0.0616; week 12: *p* = 0.2380; Fig. 2A). Two individuals in groups 2–4 exceeded published reference limits, and several had values above the population mean of 101.4 pg/mL.

NfL showed a significant group difference at week 4 (ANOVA, *p* = 0.0095), with trends toward significance at weeks 1 and 12 (*p* = 0.0917 and *p* = 0.0707). Post-hoc analyses demonstrated higher NfL in groups 2–3 (*p* = 0.0040) and group 4 (*p* = 0.0177) compared with group 1. No difference was found between groups 2–3 and group 4 (*p* = 0.9182; Fig. 2B). All values fell within published reference ranges, although several were markedly above the population mean (8.1 pg/mL)(Lanz et al., 2025).

### 3.3 CMAP responses

CMAP amplitudes were generally symmetric between sides in the frontalis, trapezius, and nasalis muscles (Table 2). Mean CMAP values for the left and right muscles were compared between group 1 and groups 2–3 (≤ 5 years of treatment) as well as group 4 (> 5 years of treatment). At week 12, Kruskal–Wallis test revealed no significant group differences in mean bilateral frontalis CMAP amplitudes (*p* = 0.6725). However, within group 1, mean frontalis CMAP amplitudes declined significantly from baseline to week 12, with the median decreasing from 1.2 mV to 0.6 mV following one BoNTA treatment (*p* = 0.0006).

**Table 2:** CMAP amplitudes week 12 or just before the next BoNTA injection.

CMAP amp (mV) Mean + − SD (range)	Reference (group 1, baseline, *N* = 9)	Group 1 (N = 8)	Group 2–3 (*N* = 18)	Group 4 (N = 9)
Frontalis dx	1.2 ± 0.63 (0.8–2.8)	0.75 ± 0.63 (0.30–2.2)	0.59 ± 0.30 (0.10–1.2)	0.79 ± 0.41 (0.20–1.4)
Frontalis sin	1.2 ± 0.58 (0.40–2.6)	0.70 ± 0.23 (0.50–1.2)	0.60 ± 0.2 (0.20–1.0)	0.61 ± 0.33 (0.20–1.1)
Trapezius dx	6.6 ± 1.3 (4.7–9.5)	5.2 ± 2.0 (3.0–8.7)	3.0 ± 1.3 (1.2–5.1)	4.1 ± 1.5 (1.9–5.6)
Trapezius sin	6.4 ± 2.2 (4.0–11.5)	5.1 ± 1.9 (2.7–9.0)	3.3 ± 1.1 (1.4–5.5)	4.4 ± 1.1 (3.1–6.2)
Nasalis dx	2.0 ± 0.58 (1.5–3.3)	1.9 ± 0.77 (1.1–3.1)	1.8 ± 0.53 (0.9–3.1)	2.3 ± 0.68 (1.2–3.4)
Nasalis sin	2.0 ± 0.73 (1.3–3.6)	2.0 ± 0.54 (1.4–3.0)	1.7 ± 0.62 (0.7–3.2)	2.2 ± 0.78 (1.0–3.3)

As a comparison, reference values from baseline (before the onset of BoNTA injections) are shown in the far left column for group 1.

**Fig. 3:**
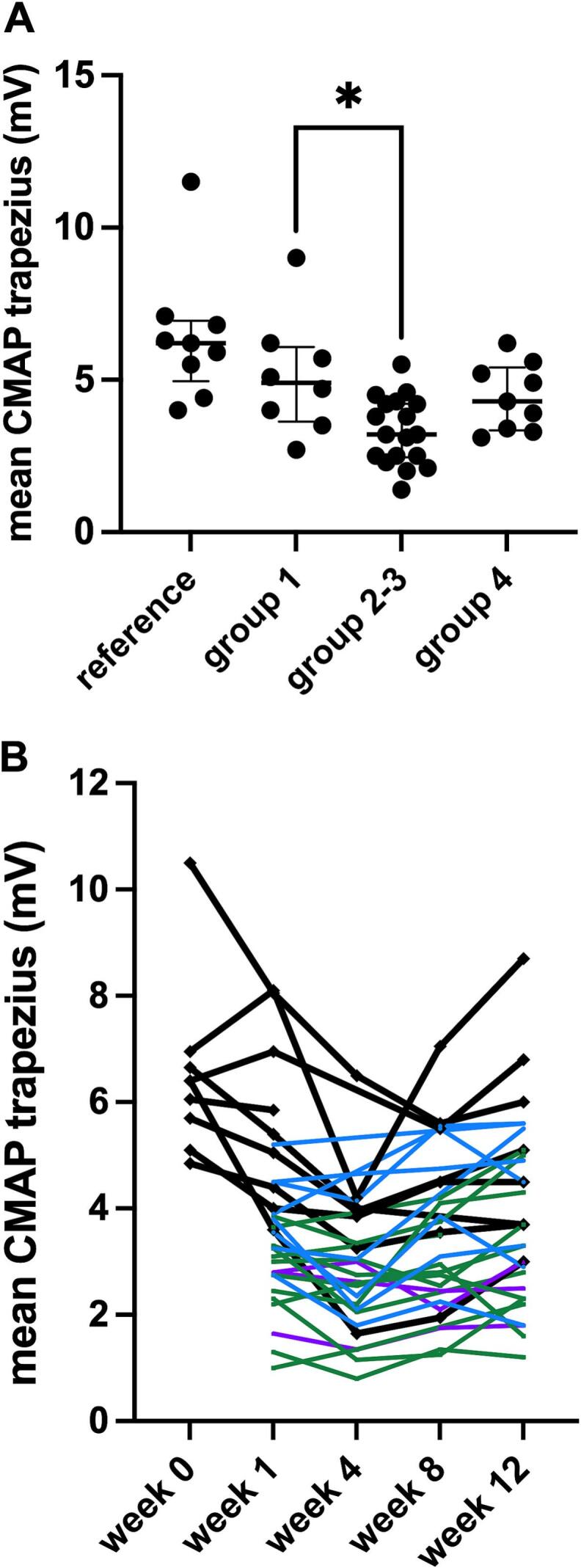
Mean CMAP amplitudes in the trapezius muscle following BoNTA exposure. (A) Mean CMAP amplitude of the left and right trapezius at baseline (reference; group 1) and at 12 weeks in groups 1 (treatment-naïve), 2–3, and 4. (B) Longitudinal mean CMAP amplitude in the trapezius muscles across study visits. Data presented as median (interquartile range). Statistical significance: *p ≤ 0.05. Black lines correspond to group 1, purple lines to group 2, green lines to group 3, and blue lines to group 4. (For interpretation of the references to colour in this figure legend, the reader is referred to the web version of this article.)

In the trapezius muscle, a significant overall change in CMAP amplitude was observed (*p* = 0.0185). Within group 1, no significant change was detected between baseline and week 12 (*p* = 0.1318; Fig. 3A). Kruskal–Wallis analyses demonstrated significant group differences in mean trapezius CMAP amplitudes (*p* < 0.0001). CMAP amplitudes were significantly lower in groups 2–3 than in group 1 (*p* = 0.0105; Fig. 3A). No significant differences were found between groups 1 and 4 (*p* = 0.1987). Several participants in groups 2–4 showed persistent failure to recover trapezius CMAP amplitude and area over time (Fig. 3B).

No group differences were observed for nasalis CMAP amplitudes (all *p* > 0.05).

## 4 EMG findings

### 4.1 Subacute neurogenic findings with ongoing denervation

Abnormal spontaneous activity consistent with ongoing denervation was frequently observed in the frontalis muscle at week 12. On the left side, abnormal spontaneous activity was detected in 25% (*N* = 2) of group 1, 60% (*N* = 9) of group 2–3, and 62.5% (*N* = 5) of group 4. On the right side, abnormal findings were present in 50% (*N* = 4), 53% (*N* = 8), and 62.5% (N = 5) in the respective groups. Despite the higher proportion of denervation signs in long-term treated groups, no significant differences in mean MUP amplitudes of the frontalis muscle were observed between groups.

In contrast, spontaneous activity in the trapezius muscle was uncommon. On the left side, abnormal spontaneous activity was present in 12.5% (*N* = 1) of group 1, 20% (*N* = 3) of patients in groups 2–3, and 0% of group 4. On the right side, no spontaneous activity was observed in group 1, compared with 20% (N = 3) in groups 2–3 and 0% in group 4. MUP amplitudes in the trapezius muscle did not differ significantly between groups at week 12.

Among the 13 patients who regularly received masseter injections (5–25 units bilaterally), EMG data were available for seven, of whom two showed abnormal spontaneous activity at week 12. No significant differences in MUP amplitudes or durations were found between injected and non-injected patients at week 1, and although a trend toward lower amplitudes was observed at week 12 among BoNTA-treated individuals, this did not reach statistical significance.

#### 4.1.1 Myopathic pattern more common in long-term BoNTA treatment

A myopathic EMG pattern was substantially more common among patients with long-term exposure to BoNTA. In groups 3 and 4, approximately 40% of patients exhibited a myopathic pattern in the frontalis muscle, and around 30% demonstrated similar changes in the trapezius muscle. These findings were observed on at least two separate examinations, including the final assessment prior to the next planned injection cycle.

In comparison, myopathic features were rare in the short-term treated groups. Only one patient in group 2 showed myopathic changes in the frontalis muscle, and no comparable findings were observed in group 1 (Table 1). This pattern suggests that cumulative or prolonged chemodenervation may contribute to structural or functional muscle alterations detectable on EMG in long-term treated patients.

### 4.2 Blink reflex

**Fig. 4:**
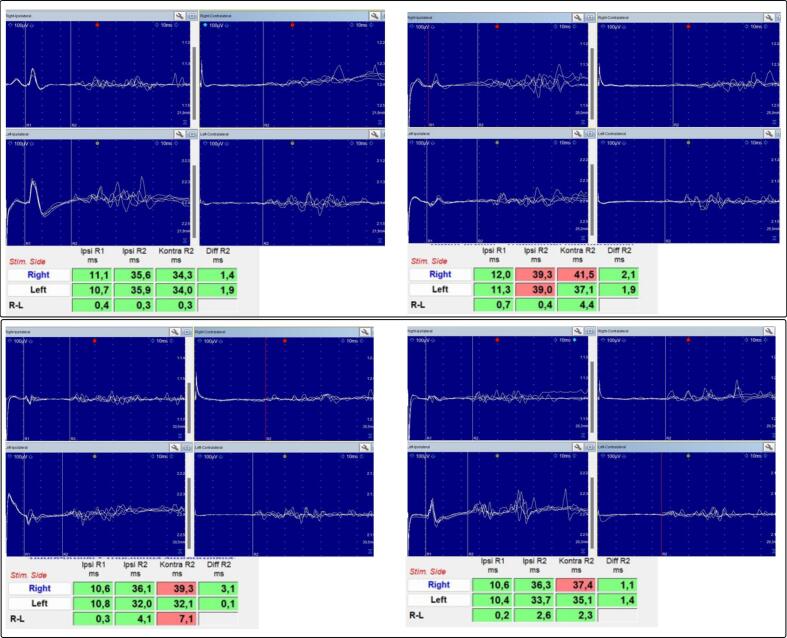
Blink reflex alterations associated with BoNTA treatment. Top panel: A treatment-naïve participant with a normal blink reflex at baseline (left) developed a prolonged contralateral R2 response 12 weeks after BoNTA injection (right). Lower panel: A participant in group 4 showing persistent prolongation of the contralateral R2 response at both 1 and 12 weeks post-injection.

All participants had normal R1 latencies (< 12 ms). Prolonged contralateral R2 latencies (> 35 ms) were observed in six participants in groups 2–4, with corresponding prolongation on infraorbital stimulation (Fig. 4). In group 1, a non-significant trend toward increased R2 latencies over time was noted. One participant in group 1 developed a prolonged contralateral R2 at week 12 (41.5 ms vs. 34.3 ms at baseline; Fig. 4).

### 4.3 CNE findings in the orbicularis oculi

**Fig. 5:**
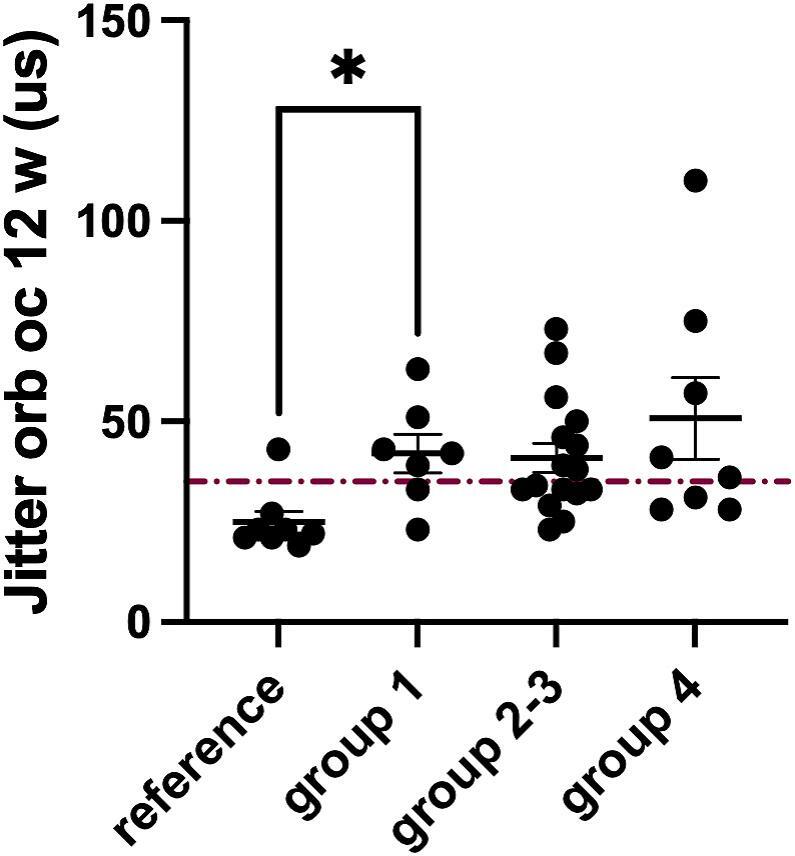
Increased jitter in the left orbicularis oculi muscle 12 weeks after BoNTA injection. Concentric needle EMG jitter values in all study groups, demonstrating that almost half of the participants—except group 1 at baseline—exhibited mean jitter values above the normal range. The red dashed line indicates the established reference limit for normal mean jitter in the orbicularis oculi muscle (35 μs). *p ≤ 0.05. (For interpretation of the references to colour in this figure legend, the reader is referred to the web version of this article.)

In group 1, mean jitter in the orbicularis oculi muscle increased significantly from baseline (25 μs; range 19–43 μs) to week 12 (42 μs; range 23–63 μs; *p* = 0.0312). Group 4 showed a trend toward higher mean jitter values (mean 51 ± 29 μs; range 28–110 μs), but between-group differences were not significant (Fig. 5). Abnormally high mean MCD values at 12 weeks were observed in 18 patients (47%): 5 in group 1 and 13 in groups 2–4 (Fig. 5).

## 5 Discussion

In this prospective study, we examined the neuromuscular and clinical consequences of long-term BoNTA treatment in women with chronic migraine. Consistent with previous evidence, BoNTA significantly reduced migraine frequency, particularly in patients with sustained treatment. However, our neurophysiological findings reveal that repeated injections are associated with persistent alterations in muscle function, including reduced CMAP amplitudes, increased spontaneous activity, and, in long-term users, a higher prevalence of myopathic EMG patterns. Importantly, electrophysiological assessments were intentionally performed at defined time points within a standard 12-week treatment cycle, with the final evaluation conducted at week 12, immediately prior to the next scheduled BoNTA injection. This time point does not represent treatment initiation, but rather the end of an active treatment cycle, when pharmacological effects are presumed to be waning, and reinjection is routinely considered in clinical practice. The persistence of electrophysiological abnormalities at week 12, therefore, suggests that signs of chemodenervation may outlast the expected therapeutic window and may accumulate across repeated cycles, despite ongoing clinical benefit. Therefore, these results indicate cumulative chemodenervation effects that may extend beyond the expected duration of pharmacological action.

### 5.1 Clinical efficacy, migraine burden, and neuromuscular side effects

The observed reduction in migraine days aligns with data from the PREEMPT trials and subsequent clinical studies demonstrating BoNTA's efficacy in chronic migraine. Notably, the greatest benefit was seen in patients with longer treatment durations, while pain intensity and quality-of-life scores remained stable across groups. This emphasizes that frequency-based indices may underrepresent clinically meaningful improvements and suggests that long-term BoNTA therapy provides sustained disease-modifying effects. The high proportion of neuromuscular side effects in long-term treated patients also highlights the importance of considering cumulative exposure when interpreting clinical outcomes.

### 5.2 CMAP reductions and evidence of cumulative chemodenervation

Motor nerve conduction studies demonstrated reductions in CMAP amplitudes in both the frontalis and trapezius muscles; however, the temporal patterns differed between muscles. In the frontalis muscle, CMAP amplitudes declined early, as evidenced by a significant within-group reduction from baseline to 12 weeks in group 1, without corresponding significant between-group differences. This suggests an early, treatment-related effect rather than progressive cumulative chemodenervation. In contrast, the trapezius muscle exhibited significant cumulative CMAP reduction, most pronounced in patients treated for 2–5 years, supporting evidence of progressive chemodenervation with repeated BoNTA exposure. People treated for more than six years did not show further decline, suggesting a possible plateau effect, potentially reflecting compensatory reinnervation or collateral sprouting. Persistent or incomplete CMAP recovery observed in several participants indicates reinnervation may be less robust in certain muscles, particularly the trapezius.

### 5.3 EMG evidence of ongoing denervation and structural muscle change

EMG recordings revealed progressive neuromuscular changes with longer treatment duration. Ongoing denervation was common in the frontalis muscle, even in patients with many years of treatment, suggesting repeated cycles of chemodenervation and reinnervation. In contrast, denervation was rare in the trapezius, which may reflect anatomical and dose-related differences.

Importantly, myopathic EMG patterns were significantly more common in long-term treated patients, appearing in roughly 40% of frontalis and 30% of trapezius muscles in groups 3 and 4. This pattern, detected on at least two occasions during the treatment cycle, suggests chronic structural remodeling. These observations raise concerns that prolonged BoNTA exposure may lead to cumulative muscle atrophy or fiber-type transformation, in line with earlier histological reports in animal and human tissues (Fortuna et al., 2015; Kaya et al., 2020).

### 5.4 Impaired neuromuscular transmission is very common upon BoNTA injections

CNE jitter increased significantly after BoNTA in treatment-naïve patients and tended to be higher in long-term-treated individuals, with abnormally high mean MCD in almost half of patients at week 12 in the non-injected orbicularis oculi muscle. Although group differences did not reach statistical significance, the increased jitter in both early and chronic phases supports the interpretation that BoNTA induces a lasting impairment of neuromuscular transmission. These findings agree with prior studies showing prolonged abnormalities in neuromuscular transmission that can persist months after clinical effects have resolved(Punga et al., 2016; Punga and Liik, 2020).

### 5.5 Serum biomarkers of axonal damage

Serum GFAP levels remained stable across treatment groups, suggesting that neither astroglial activation nor CNS injury is likely to occur in association with BoNTA treatment in migraine patients. In contrast, NfL showed a transient but significant elevation in individuals with longer treatment exposure, with the highest levels observed at week 4 post-injection. Although all values remained within published age-adjusted reference ranges, the relatively higher NfL levels in long-term-treated patients may reflect mild subclinical axonal stress or denervation. Because advancing age is associated with physiologically higher GFAP and NfL concentrations, part of this elevation may also reflect age-related variation rather than treatment effects alone.

The temporal profile, characterized by a week-4-peak followed by a decline by week 12, suggests a dynamic, reversible process possibly linked to known BoNTA mechanisms, such as transient disruption of axonal transport or synaptic remodeling. Larger longitudinal studies with age-stratified analyses are needed to determine whether NfL could serve as a sensitive biomarker for monitoring cumulative or long-term neuromuscular effects of BoNTA treatment.

### 5.6 Central sensory modulation and blink reflex changes

Blink reflex assessments revealed subtle alterations in brainstem excitability following long-term BoNTA treatment, reflected by a tendency toward prolonged contralateral R2 latencies in the blink reflex(Valls-Sole, 2019). Although these findings did not reach statistical significance, the observed pattern aligns with prior evidence indicating that BoNTA modulates nociceptive transmission not only peripherally at the NMJ but also centrally, including the trigeminal and facial pathways, supporting the concept of both peripheral and central mechanisms of action in migraine prophylaxis(Bagues et al., 2024; Nemanic et al., 2024).

In contrast to studies quantifying blink reflex magnitude using area-under-the-curve (AUC) measures (Cabib et al., 2014; Sebastianelli et al., 2023), the present analysis focused on R1 and R2 latencies. Reliable AUC quantification was not feasible in this study, as the recordings were acquired using clinical EMG software that did not support automated or standardized AUC measurements. Manual post-hoc integration was deemed insufficiently robust for consistent analysis across subjects and time points. Latency measures, therefore, represented the most reliable and reproducible blink reflex parameters available in this longitudinal clinical dataset. Future prospective studies should incorporate acquisition and analysis pipelines that enable automated quantification of blink reflex magnitude and morphology.

### 5.7 Clinical implications and considerations for long-term treatment

While BoNTA is widely regarded as safe, our study suggests that long-term and repeated injections may lead to persistent neuromuscular alterations, even in clinically asymptomatic patients. These include sustained reductions in CMAP amplitudes, increased denervation signs on EMG, higher prevalence of myopathic patterns and elevated NfL levels indicative of axonal stress. Clinicians should be aware of these cumulative changes, particularly in patients receiving high doses or injections targeting facial and neck muscles. Considerations may include adjusting injection patterns, balancing dosing across muscle groups, or incorporating periodic neurophysiological evaluation in long-term users.

### 5.8 Future directions and limitations

This study offers novel insights into the neuromuscular effects of long-term BoNTA treatment in chronic migraine; however, some limitations should be noted. The sample size was modest, and the inclusion of only women limits generalizability. As an observational study, causal relationships cannot be firmly established, and neurophysiological measurements restricted to a single injection cycle may not capture longer-term patterns of denervation or reinnervation. Furthermore, potential variability in optional injections across treatment cycles, together with incomplete longitudinal dose documentation, precluded reliable analysis of muscle-specific cumulative dose exposure. Treatment duration was therefore used as a proxy for long-term exposure, although this approach does not fully reflect muscle-specific dosing variability. Likewise, serum biomarkers were monitored only over 12 weeks, limiting interpretation of their longitudinal behavior. Future studies should include larger, mixed-sex cohorts, longer follow-up periods (potentially spanning several cycles), and assessments after treatment discontinuation. Combining electrophysiology with imaging modalities, such as muscle ultrasound or MRI, may help clarify the structural correlates of persistent denervation. Additional biomarkers of neuromuscular junction function could further enhance safety monitoring. Such integrative approaches will be essential to determine the reversibility, clinical significance, and long-term safety profile of cumulative BoNTA exposure.

## 6 Conclusions

Long-term treatment with BoNTA in women with chronic migraine is associated with sustained clinical benefit, most notably a significant reduction in migraine frequency. However, our findings also demonstrate persistent neuromuscular alterations, including reduced CMAP amplitudes, increased spontaneous EMG activity, myopathic patterns on long-term assessment, and transient elevations in serum neurofilament light chain levels, particularly in patients with prolonged exposure. These results suggest that repeated BoNTA injections may lead to cumulative chemodenervation effects, primarily in injected muscles such as the trapezius. In addition, abnormalities in neuromuscular transmission were observed in some non-injected muscles, including the orbicularis oculi. While BoNTA remains an effective and generally well-tolerated therapy, these observations underscore the importance of monitoring neuromuscular function and side effects in long-term users and highlight the need for further research to clarify the reversibility and clinical significance of these changes.

## Ethical publication statement

All the authors confirm that they have read the Journal's position on issues involved in ethical publication and affirm that this report is consistent with those guidelines.

## Declaration of competing interest

The authors declare that they have no known competing financial interests or personal relationships that could have appeared to influence the work reported in this paper.
